# Thirteen‐year genomic analysis (2010–2022) reveals high‐risk ICU *Pseudomonas aeruginosa* in China

**DOI:** 10.1002/mlf2.70097

**Published:** 2026-08-27

**Authors:** Jiadai Huang, Kaichao Chen, Miaomiao Xie, Weiyi Shen, Xiaoyang Ju, Yuchen Wu, Jiaping Li, Hongwei Zhou, Yonglu Huang, Yizhou Zhang, Yuanfeng Zhang, Tianmin Li, Letong Xu, Yue Sun, Fang Chen, Sheng Chen, Xin Deng, Rong Zhang, Yanyan Hu

**Affiliations:** ^1^ Department of Clinical Laboratory, Second Affiliated Hospital Zhejiang University School of Medicine Hangzhou China; ^2^ Department of Biomedical Sciences, College of Biomedicine City University of Hong Kong Hong Kong China; ^3^ Shenzhen Research Institute City University of Hong Kong Shenzhen China; ^4^ State Key Lab for Chemical Biology and Drug Discover and the Department of Food Science and Nutrition, Faculty of Science The Hong Kong Polytechnic University Hong Kong China

**Keywords:** antibiotic resistance, ICU, phage, *Pseudomonas aeruginosa*, virulence

## Abstract

*Pseudomonas aeruginosa* is a significant opportunistic pathogen, particularly prevalent in intensive care units (ICUs). Through a comprehensive genomic and phenotypic analysis of 518 ICU and healthy isolates collected over a 13‐year period (2010–2022), we found that ICU strains, despite reduced sequence type (ST) diversity, are dominated by persistent high‐risk clones, notably ST463 and ST1076. Despite possessing a lower overall prophage content, ICU isolates show a broader and more diverse repertoire of anti‐phage defense systems. This strengthened defense capacity correlates with ICU strains, demonstrating heightened resistance to phage challenge and contributing to lower historical phage exposure. Concurrently, ICU isolates harbor a significantly higher antimicrobial resistance (AMR) gene burden. Our detailed genomic analysis shows that this increased AMR is primarily driven by plasmid acquisition. Importantly, AMR genes associated with prophage elements are exclusively found in ICU isolates, highlighting their selective retention and functional contribution to resistance in this high‐pressure environment. Furthermore, specific virulence genes, including *exoU*, *pilA*, and *rhsP2*, are more prevalent in ICU strains, indicating enhanced pathogenicity. Collectively, these findings underscore a qualitative distinction in ICU *P. aeruginosa*: their dominance and persistence stem from highly adapted clones, robust anti‐phage defenses, rapid plasmid‐mediated AMR acquisition, and clinically selected prophage‐borne AMR.

## Impact statement

This comprehensive 13‐year genomic study reveals that *Pseudomonas aeruginosa* strains in intensive care units (ICUs) represent a distinct, highly adapted pathogen variant with profound clinical implications. The dominance of persistent high‐risk clones (ST463 and ST1076), combined with more prevalent anti‐phage defenses, elevated antimicrobial resistance via strategic plasmid acquisition, and increased virulence factors, creates a formidable therapeutic challenge. The exclusive presence of prophage‐associated antimicrobial resistance (AMR) genes in ICU isolates suggests selective pressure‐driven evolution in healthcare settings. These findings fundamentally reshape our understanding of *P. aeruginosa* pathogenesis in ICUs and highlight the urgent need for tailored infection control strategies, alternative therapeutic approaches beyond traditional antibiotics, and enhanced surveillance systems to combat these pathogens.

## INTRODUCTION

*Pseudomonas aeruginosa* is a Gram‐negative bacterium widely found in various environments, including soil, water, and hospital settings^1^. It uses a variety of virulence pathways to adapt to diverse environments, contributing to its pathogenicity. These pathways include biofilm formation^2^, quorum sensing (QS)^3^, motility^4^, the Type III secretion system (T3SS)^5^, the Type VI secretion system (T6SS)^6^, pyocyanin production^7^, *P. aeruginosa* pathogenicity islands (PAPIs)^8^, and lipopolysaccharide (LPS)^9^. The regulation of all these virulence pathways is under the control of a complex regulatory network in *P. aeruginosa*. For example, QS is a cell–cell communication system that regulates the expression of various virulence factors, including biofilm formation, toxin secretion, and motility. Motility facilitates bacterial movement and colonization mediated by flagella and type IV pili (T4P), contributing to biofilm formation and host cell attachment^10^. T3SS and T6SS are secretion systems that deliver toxic effectors into host cells and competing bacteria. Furthermore, PAPI‐1 is a genomic island found in the PA14 strain that encodes a variety of virulence factors, including those involved in adhesion, toxins, and antibiotic resistance.

*P. aeruginosa* is considered an opportunistic pathogen responsible for a wide range of infections acquired in critically ill patients in intensive care units (ICUs). It poses a significant threat to immunocompromised individuals, such as patients with cancer, burn wounds, diabetes, and cystic fibrosis^11^. This organism is associated with nosocomial infections such as gastrointestinal, respiratory, urinary tract, and bloodstream infections^12^. Treating infections caused by *P. aeruginosa* is particularly challenging due to its innate resistance and its ability to acquire additional resistance mechanisms^13^. In clinical settings, *P. aeruginosa*'s importance is underscored by its role as a leading cause of healthcare‐associated infections, contributing to prolonged hospital stays, increased morbidity, and higher healthcare costs^14^. China Antimicrobial Surveillance Network shows that *P. aeruginosa* ranks second among non‐fermenting bacteria in clinical isolation rates, further emphasizing its clinical significance^15^. While healthy individuals can also harbor *P. aeruginosa*, it typically remains asymptomatic and less prevalent. However, these carriers can serve as reservoirs, potentially spreading the bacterium in healthcare environments and complicating infection control efforts. Numerous studies have focused on the epidemiology and resistance patterns of *P. aeruginosa* in patients with weakened immune systems. For instance, high‐risk clones, including ST111, ST175, and ST235, have been identified to be responsible for epidemics of nosocomial infections^16^. Additionally, ST233, ST244, ST357, ST308, ST277, ST654, and ST298 are also high‐risk clones worldwide, and ST235 is the most prevalent clone globally^17^. Among these clones, at least 7 STs have been reported in China, including ST235, 111, 244, 308, 277, 298, and 357. Moreover, ST463, as a high‐risk clone unique to China, has been identified for its multidrug‐resistant (MDR) trait in recent years^18^.

On the other hand, limited research exists on *P. aeruginosa* strains isolated from healthy individuals^19^. The specific genetic and phenotypic differences between *P. aeruginosa* strains from the ICUs and healthy individuals remain underexplored. In this study, we collected 518 *P. aeruginosa* strains, 100 of which were from healthy individuals and the remaining 418 were from ICU patients. We obtained fecal samples from participants attending routine annual health examinations, without age or gender restrictions. These individuals were designated as the healthy cohort. Through whole‐genome sequencing (WGS) and subsequent analysis, we investigated the mechanisms that contribute to the distinct characteristics of *P. aeruginosa* isolates from ICU patients and healthy individuals in China. Specifically, we investigated the epidemiology, genetic diversity, and virulence profiles, with a particular focus on understanding the long‐term persistence of high‐risk clones like ST463 and ST1076 in ICU settings. Recognizing the role of phage–host interactions in bacterial adaptation, we analyzed the prophage content and anti‐phage defense systems in these strains, assessing their relationship with phage resistance. Furthermore, we evaluated antimicrobial resistance (AMR) differences, elucidating roles of plasmids and the selective retention of prophage‐borne antimicrobial resistance genes in shaping the resistome of ICU isolates. This integrated approach aims to provide deeper insights into the evolutionary pressures shaping *P. aeruginosa* in clinical environments.

## RESULTS

### ST463 and ST1076 emerge as persistent clonal lineages predominantly associated with the ICU environment

To understand the epidemiology and genomic diversity of *P. aeruginosa*, we collected and analyzed 518 isolates (Table S1) from ICU patients and healthy individuals over 13 years (2010–2022) in China, 377 of which were from Zhejiang Province (nine cities). The years 2021 and 2020 contributed the most samples, with 104 and 90 isolates, respectively. A total of 277 fecal samples were collected, 100 of which were isolated from healthy individuals. We also collected 141 sputum samples, 44 blood samples, 37 urine samples, and 4 central venous catheter (CVC) samples from ICU patients. Additionally, we isolated 15 *P. aeruginosa* strains from ascites (1), secretion (3), lavage fluid (1), cerebrospinal fluid (1), pus (7), and drainage fluid (2).

**Figure 1:**
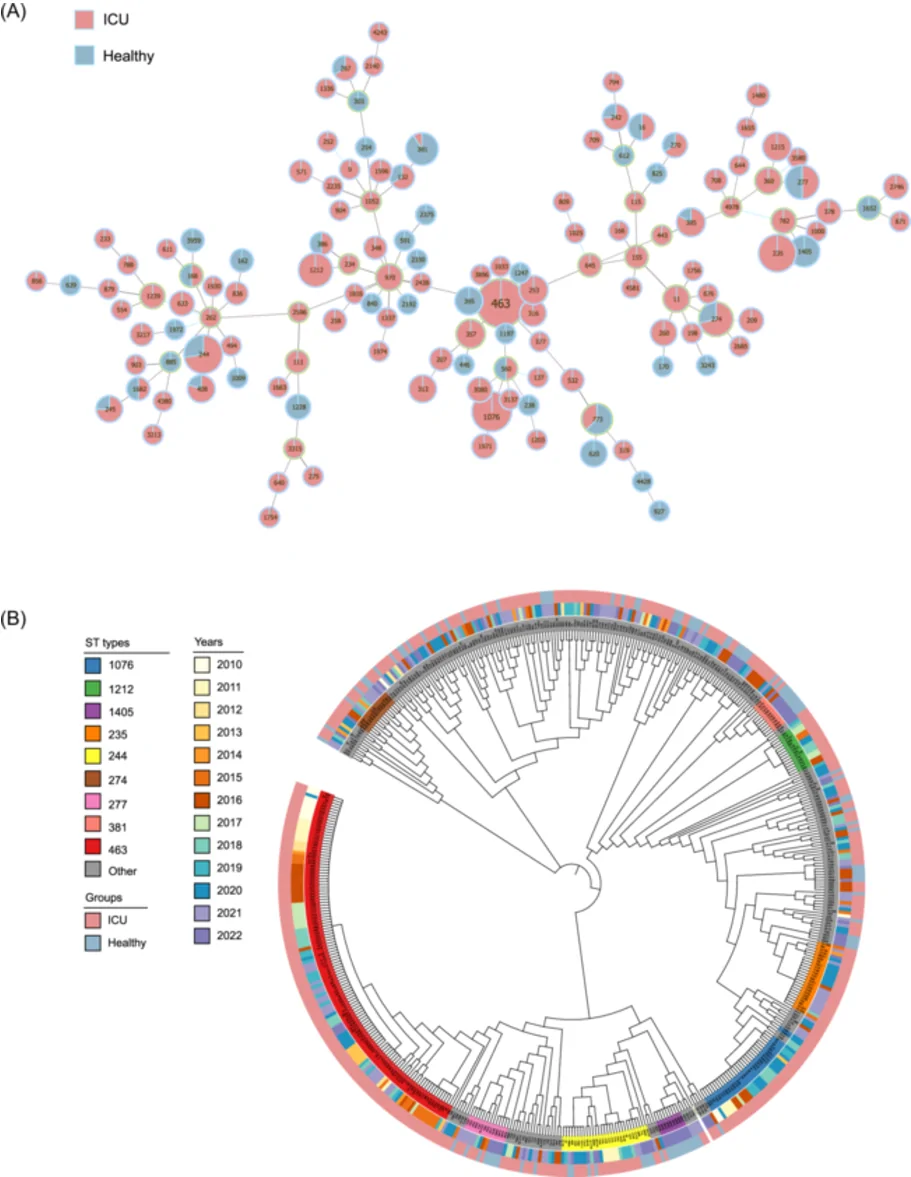
MST and phylogenetic analysis based on the clinical isolated strains of *Pseudomonas aeruginosa*. (A). MST illustrates the distribution of STs between healthy and ICU samples. Each node represents an ST, with blue nodes indicating STs from healthy samples and red nodes indicating STs from ICU samples. Node size corresponds to the number of isolates, and edges represent genetic similarities. (B). The phylogenetic tree based on core‐SNP elucidates the genetic relationships among these strains. The innermost ring represents the ST types. The middle ring indicates the isolation year. The outermost ring represents the host group. ICU, intensive care unit; MST, minimum spanning tree; SNP, single‐nucleotide polymorphism; ST, sequence type.

To investigate the genetic relationships and potential transmission pathways of *P. aeruginosa*, we performed a minimum spanning tree (MST) based on multilocus sequence typing (MLST) data from this clinical dataset (Figure 1A). Each node in the MST represents a unique sequence type (ST), with the node's size proportional to the number of isolates of that ST. A whole‐genome single‐nucleotide polymorphism (SNP)‐based phylogenetic tree was also constructed to elucidate the genetic relationships among these bacterial isolates, integrating information on STs, isolation year, and host groups (Figure 1B). ST463 and ST1076 corresponded to the two largest nodes in MST, constituting the majority of isolates and forming distinct clades within the phylogeny, indicating their significance in local pathogenesis and clonal expansion.

Within the dominant ST463 and ST1076 clades, isolates from various years were interspersed throughout the phylogenetic tree rather than forming year‐specific subclusters, indicating the long‐term persistence and circulation of these specific lineages over more than a decade. The lack of temporal structuring within these clades suggested that genetic relatedness, rather than isolation year, determined clustering patterns. This implies that these dominant clones have maintained a sustained presence in the ICU environment rather than representing repeated independent introductions. Critically, the predominant ST463 and ST1076 clades were almost exclusively comprised of isolates from ICU patients, further supporting a strong epidemiological link between these persistent lineages and the ICU setting. In contrast, isolates from healthy individuals did not form any distinct, large clusters, aligning with the general observation of lower prevalence of specific clones in healthy populations.

Moreover, we summarized the diversity differences in ST types between ICU and healthy individuals (Figure S1A). In this study, ST diversity was defined as the proportion of distinct STs relative to all isolates with an assigned ST within each group. The healthy group harbored 45 distinct STs among 95 typed isolates (47.3%), whereas the ICU group harbored 105 distinct STs among 409 typed isolates (25.7%), indicating a lower ST diversity among ICU isolates (Figure S1B). This observation aligns with previous studies and is likely attributable to antibiotic use and the critical conditions of patients^20^. In summary, the results highlight the clonal nature of the bacterial population, with ST463 and ST1076 emerging as persistent lineages predominantly associated with the ICU environment. These findings underscore the importance of ongoing genomic surveillance to track the epidemiology and evolution of these prevalent healthcare‐associated clones.

### Pan‐genome analysis reveals an adaptive accessory genome enriched for antibiotic resistance in ICU isolates

Genes do not function in isolation; rather, they form complex interaction networks that collectively give rise to phenotypes. Essential genes are critical for growth and survival, while non‐essential genes contribute more toward adaptation to different circumstances or stressors. To characterize the genetic diversity and functional characteristics of these clinically isolated *P. aeruginosa*, we conducted a pan‐genome analysis of 518 isolates together with three model strains, including PAO1 (laboratory model), PA14 (hypervirulent/acute infections), and LESB58 (cystic fibrosis epidemic strain/chronic infections). These references enabled comprehensive functional annotation and genomic contextualization of our clinical isolates. We characterized the pan‐genome into core, soft‐core, shell, and cloud genes, where core genes are present in all genomes, soft‐core genes are present in at least 95% of genomes, shell genes occur in 15%–95% of genomes, and cloud genes are found in fewer than 15% of genomes^21^. This analysis revealed the relationship between the conserved core genome, which encodes essential genes critical for basic cellular functions, and the highly variable accessory genome, which contributes to this pathogen's adaptability and pathogenicity (Figure S1C). In this pan‐genome, almost 78% of genes belonged to accessory genomes, while core and soft‐core genes accounted for only 16.7%. Further, the enrichment analysis of core genes based on Gene Ontology (GO) and Kyoto Encyclopedia of Genes and Genomes (KEGG) databases^22^ identified several pathways enriched in fundamental cellular processes, including cellular protein metabolic process, ribosome, and translation (Figure S1D).

In contrast, diverse cloud genes contributed to the genomic plasticity of these isolates (Figures S1E–F). Comparison of GO terms enriched in healthy and ICU groups revealed that healthy isolates were predominantly associated with cell surface structure and core metabolism, such as the LPS biosynthetic process. Concurrent enrichment of DNA maintenance, and diverse oxidoreductive and catalytic activities indicates robust genome stability mechanisms and energy metabolism, consistent with a bacterial community adapted to a relatively stable, growth‐supporting physiological state. In contrast, the ICU group showed enrichment of functions linked to environmental stress adaptation, notably “response to antibiotic” and “transmembrane transporter activity”, consistent with selection for antibiotic resistance and enhanced efflux or transport capacity under intensive antimicrobial exposure in the ICU setting^23^.

### ICU isolates harbor fewer prophages and show reduced historical phage exposure compared to healthy isolates

**Figure 2:**
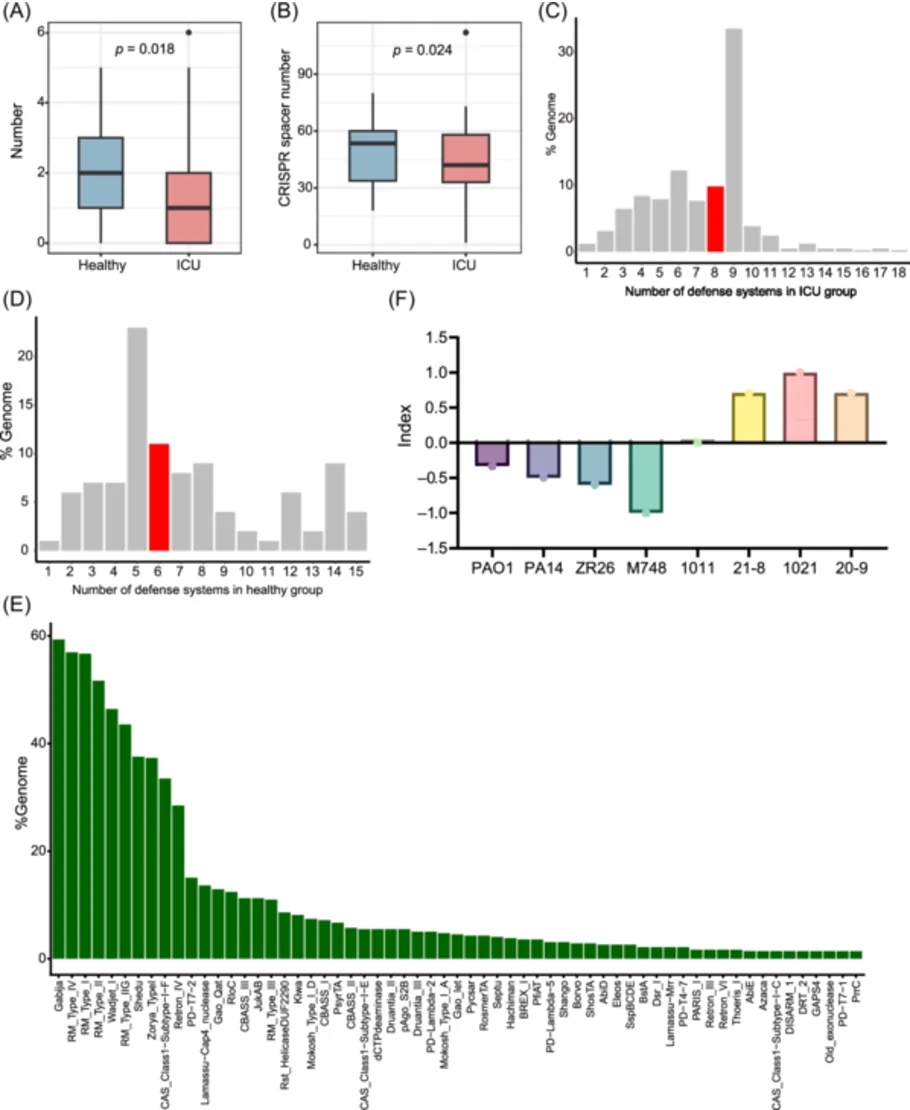
Prophage content and defense system analysis on healthy and ICU samples. (A) Prophage content comparison between healthy and ICU samples. Significance was determined by the Wilcoxon test, *p* = 0.018. (B) Phage exposure between healthy and ICU samples. Significance was determined by the Wilcoxon test, *p* = 0.024. (C, D) Number of defense systems per genome in *P. aeruginosa* strains from ICU patients (C) and healthy individuals (D). The median number is shown in red. (E) Diversity of defense systems found in the genomes of *P. aeruginosa* strains from the ICU group, ordered and colored in a gradient of abundance. The *X*‐axis represents the different types of defense systems. (F) Phage interaction index for *P. aeruginosa* strains. Index values closer to 1 represent a stronger ability to infect others and resist infection, while values closer to –1 represent higher susceptibility to infection and weaker resistance.

Building on insights into pan‐genome diversity and the adaptive role of mobile genetic elements (MGEs), we investigated the dynamic interplay between bacteria and phages by identifying and characterizing prophages and historical phage exposure in isolates from healthy individuals and ICU patients. Using DEPhT^24^ for prophage prediction, followed by quality assessment with CheckV^25^, a total of 833 prophages were identified across 398 isolates. Quality assessment revealed 227 complete, 49 high‐quality, 460 medium‐quality, and 97 low‐quality prophages (Table S2). Our analysis revealed that isolates from healthy individuals harbored a significantly higher number of prophages compared to those from ICU patients (Wilcoxon test, *p* = 0.018) (Figure 2A). Furthermore, analysis of CRISPR (Clustered Regularly Interspaced Short Palindromic Repeats) spacer content showed that healthy isolates possessed a significantly greater number of spacers than ICU isolates, suggesting distinct histories of phage exposure between the two groups (Wilcoxon test, *p* = 0.024) (Figure 2B).

### ICU isolates possess a richer and more diverse anti‐phage defense repertoire

Given the observed differences in prophage content and phage susceptibility, we hypothesized that these phenotypic variations in phage susceptibility are mediated by differences in the bacterial anti‐phage defense system repertoire^26^. To test this hypothesis, we analyzed bacterial anti‐phage defense systems using DefenseFinder^27^. Examination of the per‐genome distribution of defense system counts revealed distinct patterns between the two groups (Figure 2C,D). Specifically, ICU isolates showed a higher median number (8) of defense systems, while healthy isolates displayed a lower median (6), suggesting that ICU isolates generally possess a richer anti‐phage repertoire, potentially contributing to their reduced phage susceptibility.

A detailed analysis of the specific defense system types present in ICU isolates revealed a broader spectrum of anti‐phage mechanisms (Figure 2E) than those of healthy individuals (Figure S2A). Among the most prevalent defense systems detected in ICU isolates were Gabija, various restriction–modification (RM) systems, and diverse CRISPR‐Cas subtypes. The varying frequencies of these systems indicate a heterogeneous landscape of anti‐phage strategies in the ICU environment. We further investigated the distribution of defense systems within the major STs in the ICU environment (Figure S2B). This analysis revealed that ST463 isolates harbored a statistically higher number of defense systems than ST1076 (Dunn's test, *p* < 1.0 × 10^−18^) and other ST types (Dunn's test, *p* < 3.3 × 10^−31^) in the ICU.

### ICU‐associated phages encode broader anti‐defense repertoires and show enhanced infectivity against healthy‐environment strains

Complementing our analysis of bacterial defense mechanisms, we investigated the distribution of anti‐defense systems, which are typically encoded by phages to counteract host defenses. Genomic analysis (Figure S2C,D) revealed that ICU isolates harbored a significantly higher prevalence and broader range of phage‐encoded anti‐defense mechanisms compared to healthy isolates. Specifically, comparative analysis (Figure S2E) showed an enrichment of anti‐defense systems such as AcrIF subtypes in ICU isolates, with the exception of AcrIF12, which was enriched in healthy isolates.

To functionally assess the virulence of phages associated with these environments, we performed plaque assays using bacterial supernatants from eight strains: two ST463 isolates (#1011, #21‐8), two ST1076 isolates (#1021, #20‐9), and four comparative strains, including ZR26 (ICU), M748 (healthy carrier), PAO1, and PA14. Using a pairwise cross‐infection assay among the eight strains, we quantified each isolate's ability to produce phages that infect other hosts (“phage‑out”) and its susceptibility to phages produced by the other isolates (“phage‑in”) (Figures S2F,G). Supernatants from ST463 and ST1076 isolates consistently generated higher plaque counts than the other strains, particularly than healthy‐carrier and reference strains. To summarize these reciprocal interactions, we calculated a phage interaction index for each strain, defined as (phage‑out − phage‑in)/(phage‑out + phage‑in) (Figure 2F). It was found that a heightened capacity of phages associated with dominant ICU STs to infect and lyse strains from healthy environments, while ICU isolates showed strong resistance to phages originating from the same environments. Notably, prophages from ST463 and ST1076 appeared to have a broad host range (Figure 2F). This suggests that prophages harbored by these dominant clones have evolved the capacity to recognize a wider repertoire of cell surface receptors or deploy more effective anti‐defense mechanisms, thereby expanding their host range among diverse *P. aeruginosa* populations and enhancing their ecological fitness in competitive environments.

### Plasmids drive AMR acquisition in ICU isolates

**Figure 3:**
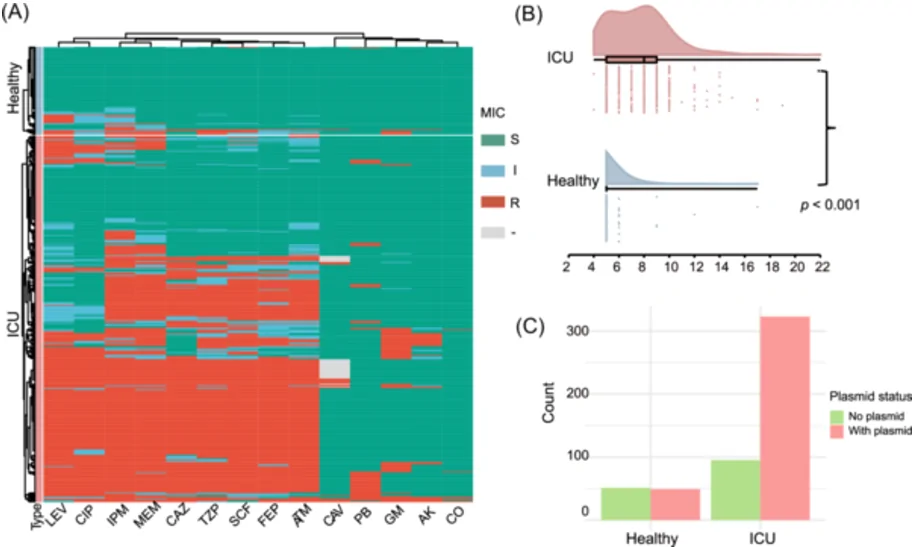
Profiling of AMR genes in healthy and ICU groups. (A) Heatmap of phenotypic antimicrobial susceptibility profiles based on minimum inhibitory concentration (MIC) testing. Antibiotics: polymyxin B (PB), gentamicin (GM), clavulanic acid (CAV), amikacin (AK), colistin (CO), levofloxacin (LEV), ciprofloxacin (CIP), imipenem (IPM), meropenem (MEM), ceftazidime (CAZ), aztreonam (ATM), piperacillin‐tazobactam (TZP), cefepime (FEP), and cefoperazone‐sulbactam (SCF). Red, blue, and green indicate resistant (R), intermediate (I), and susceptible (S) strains, respectively, and gray indicates missing data. (B) Raincloud plot shows that ICU groups carried more AMR genes than healthy groups. Significance was determined by the Kolmogorov–Smirnov test, *p* < 0.001. (C) Plasmid carrying in the healthy and ICU groups.

To assess the AMR landscape, we first performed phenotypic susceptibility testing by determining the minimum inhibitory concentrations (MICs) of 14 antibiotics across all isolates. MIC values were interpreted according to clinical breakpoints and classified as resistant (R), intermediate (I), or susceptible (S). ICU isolates showed higher resistance rates across most tested antibiotics, particularly to carbapenems and extended‐spectrum cephalosporins, compared to healthy‐carrier isolates (Figure 3A). To identify the genetic basis of this resistance, we identified all AMR genes using AMRFinder^28^ and subsequently quantified plasmid content using Plasmer^29^. Comparison of AMR gene counts between ICU and healthy isolates revealed that ICU isolates carried significantly more AMR genes than healthy isolates (Kolmogorov–Smirnov test, *p* < 0.001) (Figure 3B and Table S3). Concurrently, Plasmer analysis revealed a higher prevalence of plasmids in ICU isolates than in healthy isolates (Figure 3C).

To investigate the genomic locations of these AMR genes and understand their differential contributions, we further partitioned the genome sequences into chromosome, plasmid, and prophage fractions and then re‐identified AMR genes within each fraction. Crucially, intrinsic AMR genes associated with the reference strain PAO1 were excluded from this analysis to highlight clinically acquired resistance profiles in the clinical isolates. This detailed analysis highlighted that the increased AMR burden observed in ICU isolates was predominantly attributable to genes located on plasmids (Figure S3A). While chromosomal AMR genes (Figure S3B) showed less pronounced differences between groups, plasmid‐borne AMR genes, such as those encoding *Klebsiella pneumoniae* carbapenemase (KPC), were significantly enriched in ICU isolates. KPC hydrolyzes a wide range of β‐lactams, including carbapenems, and KPC‐encoding genes disseminate rapidly worldwide^30^. Notably, the *bla*_KPC‐2_ gene was nearly universally carried by ST463 isolates and was also prevalent among ST1076 isolates in the ICU group. This strongly suggests that plasmids serve as the principal vehicle for the acquisition and dissemination of resistance determinants in the ICU environment, driving much of the observed AMR difference.

Moreover, this analysis extended to the association of AMR genes with prophages. Although ICU isolates harbored fewer prophages overall (Figure 2A), AMR genes associated with prophage elements were exclusively detected in ICU isolates (Figure S3C), suggesting that prophages in ICU isolates are subject to strong positive selection pressure to retain genes that confer a critical survival advantage, such as AMR genes.

### ICU isolates are enriched for hypervirulence‐associated genes, with ST‐specific virulence strategies

**Figure 4:**
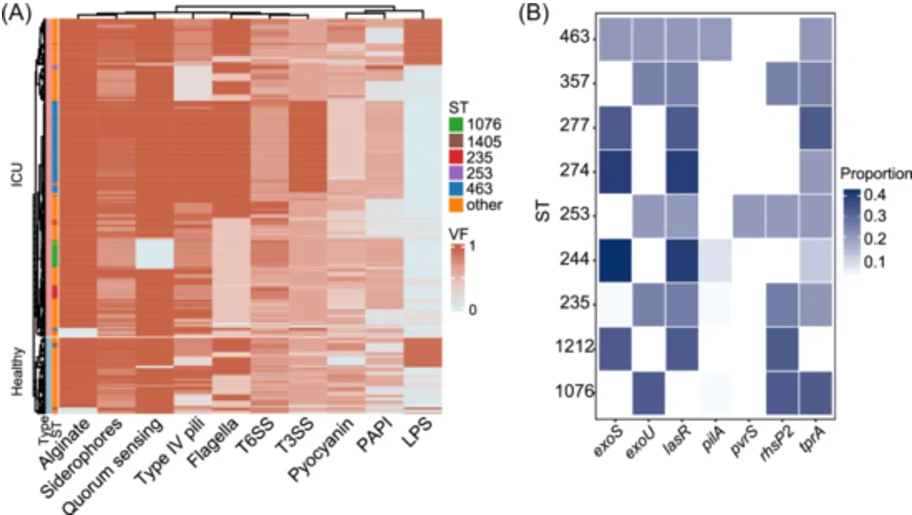
Distribution of virulence‐related genes in clinically isolated *P. aeruginosa*. (A) The heatmap illustrates the distribution of virulent pathways across healthy and ICU samples. Rows represent individual samples and columns represent different virulence factors such as alginate, siderophores, quorum sensing, type IV pili (T4P), flagella, Type VI secretion system (T6SS), Type III secretion system (T3SS), pyocyanin, and lipopolysaccharide (LPS). The color intensity indicates the abundance of each pathway. (B) The heatmap visualizes the relationships between major ICU STs and key virulence genes such as *exoS*, *exoU*, *lasR*, *pilA*, *pvrS*, *rhsP2*, and *tprA*. ICU, intensive care units.

To elucidate the virulence potential of these strains, we analyzed the distribution and prevalence of virulence‐related genes in isolates from ICU patients and healthy individuals. Virulence genes were categorized into several functional pathways, including alginate production, siderophore synthesis, QS, T4P, flagella motility, T6SS, T3SS, pyocyanin production, PAPI, and LPS biosynthesis (Figure 4A and Table S4). While the overall number of virulence‐related genes did not differ significantly between groups (Figure S4A), distinct patterns were observed at the level of specific pathways and individual genes (Figures 4A and S4B).

Notably, specific virulence genes, including *exoU*, *pilA*, *rhsP2*, and *tprA*, were differentially represented between the ICU and healthy isolates (Figure S4B). For example, both groups tended to harbor *exoS*, while ICU isolates preferentially carried *exoU*. Both ExoU and ExoS are effector proteins secreted by the T3SS, enabling *P. aeruginosa* to inject effectors directly into host cells. ExoU is recognized as a marker of hypervirulence, whereas ExoS is not consistently associated with increased virulence^31^. Further analysis revealed that genes associated with PAPI‐1, such as *tprA*, were more frequently found in ICU isolates. Additionally, the *pilA* gene, involved in bacterial adherence^32^, and the *rhsP2* gene, related to T6SS^33^, showed higher prevalence in ICU isolates than in healthy isolates.

The distribution of key virulence genes also varied among specific STs in the ICU (Figure 4B). Several high‐risk ICU STs, including ST235, ST253, ST357, ST463, and ST1076, showed a strong association with the presence of *exoU*. Specifically, ST463 isolates frequently carried both *exoU* and *exoS*. The co‐occurrence of both effectors in a single strain enhances its pathogenic arsenal, making it a formidable pathogen in clinical settings, as previously reported^34^. Regarding PAPI‐1, ST253 was the only ST observed to harbor complete PAPI‐1 gene clusters among all analyzed strains. Furthermore, the absence of QS genes in ST1076, particularly those of the *las* system, suggests an alternative virulence strategy. Strains lacking the *las* system but retaining virulence have been isolated from clinical settings and environments^35^. Despite this absence, the persistence of ST1076 in clinical settings suggests that it compensates through other alternative mechanisms, potentially involving other signaling pathways.

### Pyocyanin production reflects ST‐specific virulence potential

**Figure 5:**
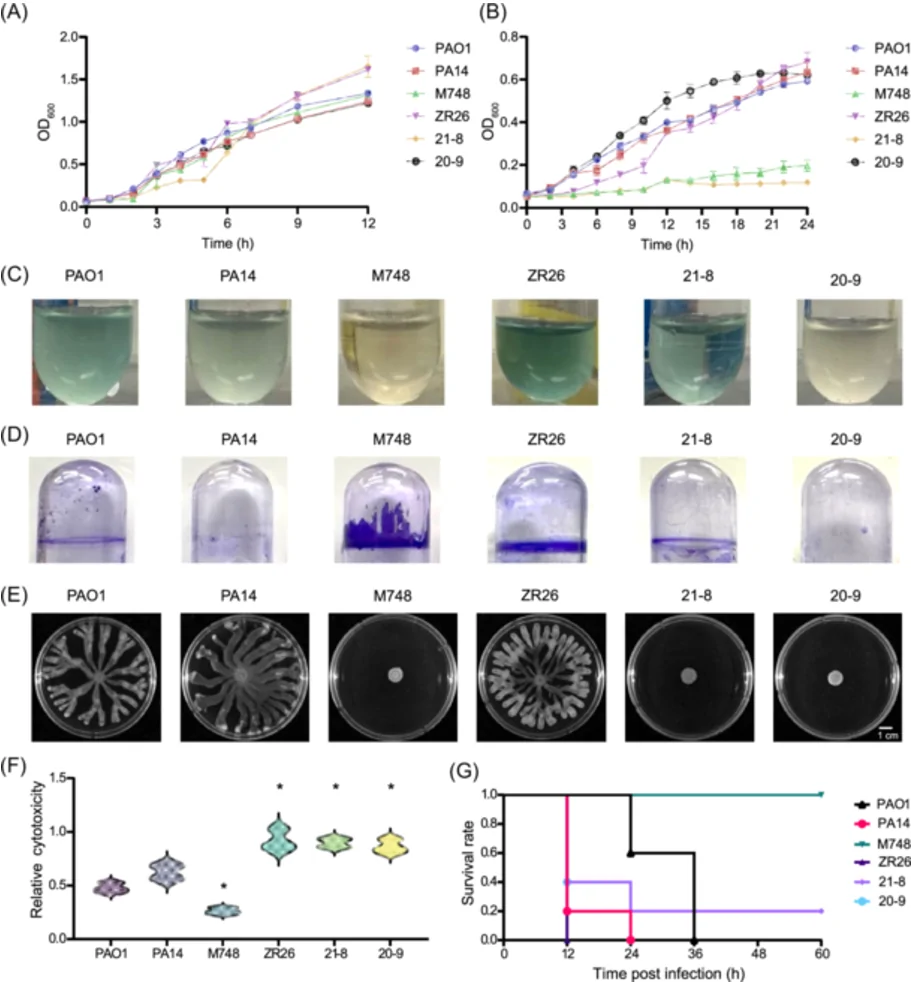
Phenotype experimental validation of representative isolates. (A, B) Growth curve measured in LB medium (A) and in M9 medium (B). (C) Pyocyanin detection assay. The green color intensity corresponds to the concentration of pyocyanin produced. (D) Biofilm detection assay results, The color intensity corresponds to the biomass of the formed biofilm (crystal violet staining). (E) Swarming motility assay results, showing the motility zones of representative strains. (F) Relative cytotoxicity of representative isolates. (G) Survival rate of the mice infected with the representative isolates. All the error bars used were obtained from at least two biological and technical replicates. The values represent mean ± standard deviation (SD). The asterisk (*) indicates a statistically significant difference compared to PAO1 (*p* < 0.05, determined by a *t*‐test).

To further investigate the genotype–phenotype relationship among those potentially hypervirulent *P. aeruginosa* STs, we conducted a series of phenotypic experiments on six selected strains, consistent with those in the plaque assays. These clinical isolates were selected based on their previously characterized hypervirulence‐associated features. We first assessed their growth kinetics in LB broth and M9 minimal medium. The results revealed no significant difference in growth among these strains in LB broth, although #21‐8 (ST463) showed a slight growth delay (Figure 5A). In M9 medium, #21‐8 and M748 (ST1405) showed growth defects, potentially attributable to their inability to utilize glycerol as a carbon source (Figure 5B).

Pyocyanin is a blue‐green pigment and virulence factor that generates reactive oxygen species, causing host cell damage and modulating immune responses. It can exacerbate the damaging effects in immunocompromised individuals by impeding wound healing through its anti‐proliferative activity^36^. Given the top role of the las system in the QS pathway in *P. aeruginosa*, we hypothesized that pyocyanin production could be affected in ST1076 isolates. As expected, the ST1076 strain (#20‐9) produced much less pyocyanin than PAO1, similar to the *lasR* mutant (Figures 5C and S5A‐B). Notably, the strain M748, which was isolated from healthy individuals, also produced decreased pyocyanin, suggesting its low virulence level. On the contrary, the production of pyocyanin increased significantly in ZR26 (ST253), indicating its potential for hypervirulence.

### ST1405 and ST253 show enhanced biofilm formation

Since biofilm formation protects bacteria from environmental stress and facilitates colonization, we performed biofilm formation assays to assess phenotypic differences among these strains. The results indicated that M748 and ZR26 showed robust biofilm formation (Figures 5D and S5C). Under general conditions, biofilm formation is inversely linked to motility through the secondary signal c‐di‐GMP^37^. We therefore assessed swarming motility, which is primary driven by flagella. As expected, M748 (ST1405) showed decreased motility compared to the model strains, consistent with its enhanced biofilm formation (Figure 5E). However, ZR26 (ST253) retained strong swarming ability while simultaneously showing enhanced biofilm formation. Moreover, #20‐9 (ST1076) showed decreased swarming motility compared to the model strains, potentially attributable to the loss of the *las* QS system.

### Cytotoxicity and mouse infection experiments confirm the hypervirulence of ICU‐associated *P. aeruginosa*

ZR26 (ST253) demonstrated strong virulence‐associated characteristics at both the genotypic and phenotypic levels. It harbored numerous virulence and AMR genes, coupled with robust phenotypic traits, including elevated pyocyanin production, enhanced biofilm formation, and strong motility (Figure 5D–E). ST463 isolates showed the potential for strong T3SS‐mediated cytotoxicity, owing to the co‐presence of *exoU* and *exoS* genes. ST1076 also harbored *exoU*, a marker of hypervirulence; yet, its other phenotypic traits were less pronounced. In contrast, M748 (ST1405) displayed comparatively weaker virulence‐associated genotypic and phenotypic characteristics, consistent with its origin from a healthy carrier. To formally validate these virulence predictions, we performed cytotoxicity assays and mouse infection experiments. Both the lactate dehydrogenase (LDH) release assay and the mouse infection model demonstrated that ICU isolates showed significantly higher cytotoxicity and virulence compared to isolates from healthy individuals and PAO1 (Figure 5F,G). These findings suggest that *P. aeruginosa* isolates from ICU patients not only possess a greater capacity to damage host cells but also show enhanced pathogenic potential *in vivo*, aligning with the clinical observations that ICU‐associated strains are more virulent.

## DISCUSSION

*P. aeruginosa* is a leading cause of nosocomial infection and poses a severe threat in hospital settings, particularly in ICUs, while also asymptomatically colonizing the intestinal tracts of healthy individuals (~3%). Given the dual ecological existence, our comparative analysis of *P. aeruginosa* isolates from Chinese ICU patients and healthy individuals uncovered distinct adaptive landscapes within the ICU cohort. These distinguishing characteristics, notably in their genetic diversity, virulence profiles, and antimicrobial resistance, carry significant implications for shaping effective prevention and treatment strategies.

Our genomic analysis revealed the clonal dominance and persistence of *P. aeruginosa* ST463 and ST1076 within Chinese ICU patients, with both clones circulating for over a decade (Figure 1). This distinct epidemiological pattern contrasts with the global prevalence of ST235 as a leading high‐risk clone^17^, suggesting unique selective pressures in Chinese ICUs. One plausible driver of this landscape is the widespread carbapenem usage in hospitals, especially in ICUs^38^. Specifically, ST463 and ST1076 isolates frequently carry *bla*_KPC‐2_/*bla*_KPC‐3_ plasmids, a key carbapenem resistance mechanism, unlike the alternative resistance strategies often seen in Chinese ST235. This selective advantage likely enables their sustained persistence and adaptation in the high‐pressure ICU environment. These findings not only delineate the unique *P. aeruginosa* epidemiology in Chinese ICUs but also underscore the critical link between antimicrobial stewardship and the emergence of dominant, resistant clones, necessitating targeted surveillance. Furthermore, the MST analysis further supported the genetic connectivity between ICU and healthy isolates (Figure 1). The translocation of endogenous intestinal *P. aeruginosa* represents one recognized route for systemic infection^39^. The presence of high‐risk clones in both groups, including ST277 and ST274, indicates that *P. aeruginosa* can cross the intestinal barrier through the intestinal epithelium under permissive conditions^40^. This capacity to invade the bloodstream and other sterile body sites underscores the importance of monitoring these clones in hospitals.

Our study also examined the intricate interactions between *P. aeruginosa* and phages, revealing distinct evolutionary trajectories shaped by the high‐pressure ICU environment. These observations strongly suggest a robust arms race between bacteria and phages in the ICU setting. The lower prophage content in ICU isolates, combined with their advanced defense capacity and reduced phage exposure (Figure 2), points toward an environment in which phage predation constitutes a potent selective force. Bacteria that survive and persist in the ICU are those that are highly adept at countering phage attacks, driving selection for strains with more numerous and diversified defense systems. Notably, the dominant ST463 isolates harbored an exceptionally high number of defense systems, likely contributing to their long‐term establishment in Chinese ICUs.

However, the finding that ICU isolates are also associated with a higher prevalence and broader range of phage‐encoded anti‐defense mechanisms (Figures 2 and S2) appears to be seemingly contradictory, where both bacterial defenses and phage counter‐defenses are enriched, underscoring the co‐evolutionary struggle in the ICUs. This implies that phages capable of infecting and lysing *P. aeruginosa* in this environment are not merely abundant, but have evolved superior lytic capabilities and effective strategies to circumvent host defenses. The co‐enrichment of bacterial defenses and anti‐defense systems thus represents a stable, high‐tension equilibrium where both sides are constantly adapting. In the era of escalating AMR, phage therapy offers a promising alternative or adjunct to conventional antibiotics, particularly against difficult‐to‐treat infections caused by pathogens like multidrug‐resistant *P. aeruginosa* ^41^. This knowledge provides a crucial foundation for the rational design and selection of effective therapeutic phages or phage cocktails. Moreover, understanding the dynamic interplay of defense and anti‐defense systems can inform strategies to mitigate the emergence of phage resistance during therapy, thereby maximizing its long‐term clinical efficacy^42^.

Due to both intrinsic and acquired resistance to a broad spectrum of antibiotics, *P. aeruginosa* ranks among the most clinically significant superbugs^43^. Our findings demonstrated a significantly higher AMR gene burden in ICU isolates compared to those from healthy individuals (Figure 3), consistent with previous studies conducted in China and the United States, that the ICU isolates had lower susceptibility rates than non‐ICU isolates^19^. Crucially, our detailed genomic analysis revealed that this heightened AMR in ICU strains was predominantly plasmid‐borne rather than chromosomally integrated. The high frequency of plasmid carriage in these isolates further corroborates this finding, as plasmids serve as primary vectors for horizontal gene transfer, facilitating the rapid dissemination of AMR genes among bacterial populations^44^.

Prophages have been reported to confer multiple fitness benefits, such as resistance to environmental stressors and access to additional genetic material^45^, ^46^. Analogous to the “plasmid paradox”^47^, while carrying prophages generally incurs an energetic cost, comparable to the maintenance burden of plasmids, their selective retention suggests a significant functional advantage in specific environments. Our finding that AMR genes associated with prophage elements were exclusively detected in ICU isolates exemplifies this principle, and is consistent with the established understanding that plasmids are the primary carriers of AMR genes, while phages harbor them far less frequently^48^. The exclusive localization of AMR genes within prophage elements of ICU strains therefore suggests that under the intense selective pressure of the ICU, a subset of prophages can acquire and stably maintain AMR genes. Despite the overall lower prophage content in ICU isolates, these AMR‐carrying prophages likely confer a critical fitness advantage under antibiotic pressure, justifying their metabolic cost and contributing to the long‐term adaptation and persistence of ICU‐associated clones.

Regarding virulence, although no significant difference was observed in the overall number of virulence‐associated genes between ICU and healthy isolates, specific genes and pathways varied (Figure 4). ICU isolates showed a higher prevalence of *exoU*, particularly among high‐risk clones, such as ST463, ST1076, ST235, and ST253. Several studies have highlighted the contribution of *exoU*‐*exoS* co‐occurrence to the emergency of high‐risk *P. aeruginosa* clones, including ST463^34^, ^49^. Additionally, ST253 carried supplementary PAPI‐1 genes, suggesting a higher virulence potential. Interestingly, ST1076 lacked the *las* QS system, which would theoretically impair biofilm formation, motility, and pyocyanin production. As expected, ST1076 showed deficiencies in all three phenotypes (Figure 5). Nevertheless, ST1076 displayed enhanced virulence in cytotoxicity and mouse infection experiments. Whether this enhanced pathogenicity results from QS rewiring or from *las*‐independent mechanisms remains unclear and warrants further investigation. These observations suggest that, beyond *exoU*, ST1076 might exploit alternative pathways to potentiate its virulence.

Several limitations should be acknowledged. First, our study mainly focused on isolates from Zhejiang Province, China, and the generalizability of our findings to other geographic regions remains to be established. While ST463 has been reported as a China‐endemic high‐risk clone in previous national surveillance studies^50^, ^51^, whether its strong ICU‐specific enrichment and associated genomic signatures are regionally specific or represent a broader national or global phenomenon requires validation in multicenter cohorts with detailed ward‐level clinical metadata. Most publicly available *P. aeruginosa* genome datasets lack ICU versus non‐ICU stratification, precluding direct comparison at present. Second, our single‐time‐point sampling from healthy populations does not allow definitive determination of stable colonization versus transient carriage. However, this limitation likely biases our analysis toward underestimating rather than overestimating differences between healthy and ICU populations, as transient carriage would introduce heterogeneity into the healthy group. The strong and statistically significant genomic and phenotypic differences that we observed, particularly the dominance of high‐risk clones in ICU settings and their distinctive prophage‐mediated interactions, suggest population‐level patterns driven by fundamentally different selective environments rather than individual colonization dynamics. Third, our comparative genomic analysis focused primarily on gene presence/absence rather than allelic variation or functional inference. While this approach enabled efficient large‐scale screening of genomic differences, it may overlook functionally important sequence variants, such as loss‐of‐function mutations in present genes or subtle allelic differences affecting protein function.

Overall, this study provides valuable insights into the epidemiology, resistance mechanisms, and virulence profiles of *P. aeruginosa* in both ICU and community contexts. Our findings highlight that the multifaceted adaptive strategies used by this pathogen, encompassing clonal dominance, enhanced defense systems, plasmid‐mediated AMR dissemination, and context‐specific virulence gene repertoires, collectively drive the disproportionate prevalence of high‐risk clones in the ICU environment. These findings underscore the need for targeted surveillance and innovative therapeutic strategies to combat these versatile and dangerous pathogens more effectively.

## MATERIALS AND METHODS

### DNA extraction and genome sequencing

All isolates were collected from 17 provinces throughout China, of which 377 isolates were obtained from 26 hospitals across 9 cities in Zhejiang Province, representing the largest single‐province collection in this study (Table S1). Genomic DNA was extracted from isolates as previously described^52^. According to standard protocols, indexed Illumina sequencing libraries were prepared using the TruSeq DNA PCR‐free Sample Preparation Kit (Illumina Inc.). These libraries were then sequenced on the Illumina HiSeq X10 platform using 150‐bp paired‐end strategies, as per the manufacturer's protocols (Bionova). Trimmed reads were assembled into contigs using SPAdes v3.11.1^53^.

### MST tree and phylogenetic tree construction

The MST tree was generated based on the seven housekeeping genes included in the *P. aeruginosa* MLST scheme (*acsA*, *aroE*, *guaA*, *mutL*, *nuoD*, *ppsA*, and *trpE*). These sequences were extracted from each isolate, aligned, and analyzed to compute allelic distances for MST construction. Core‐SNP phylogenetic trees were constructed using Snippy (version 4.6.0, https://github.com/tseemann/snippy) for variant calling against the *P. aeruginosa* PAO1 reference genome, followed by core genome alignment extraction. Approximately maximum‐likelihood trees were inferred using FastTree^54^ (version 2.1.11) with the generalized time‐reversible (GTR + CAT) model, and visualized using iTOL^55^.

### Pan‐genome analysis

The pan‐genome analysis was conducted using Roary v3.13.0^56^ with the GFF3 output from Prokka v1.14.6^57^. Initially, the genomic sequences of all isolates and three model strains (PAO1, PA14, and LESB58) were annotated with Prokka to identify features. Following annotation, Roary was used to perform the pan‐genome analysis. The analysis was performed with the default settings, and the results included the core and accessory genome content.

### GO and KEGG enrichment analysis

GO and KEGG enrichment analysis of core genes from pan‐genome analysis was carried out using the R package clusterProfile^58^. GO terms and KEGG pathways with *p* < 0.05 were considered significantly enriched.

### Plasmid prediction and identification of defense/anti‐defense systems, AMR genes, and virulence‐related genes

Plasmid prediction for the *P. aeruginosa* isolates was performed using Plasmer Docker^29^. Anti‐defense systems and defense systems were identified using DefenseFinder, and it was run with default parameters. Virulence genes were identified by aligning the genomic sequences against the virulence factors database (VFDB)^59^ using the BLAST (version 2.12.0) with default parameters^60^. Besides, AMR genes were detected using AMRFinder^28^. All related visualizations were generated using the R package ggplot2^61^.

### Prophage identification and phage exposure analysis

Prophages within the *P. aeruginosa* genomes were identified using DEPhT^24^ with its default parameters and subsequently quality‐assessed using CheckV^25^ to evaluate completeness and contamination. All prophage elements identified by DEPhT were included in the analysis, with CheckV quality metrics reported in Table S2.

To assess phage exposure, we first downloaded a comprehensive phage database from NCBI^62^. Subsequently, CRISPR spacers were identified from the *P. aeruginosa* genomes using the CRT tool^63^. Phage exposure frequency was then determined by searching for matches between bacterial CRISPR spacers and the phage sequences in the downloaded database using the SpacePHARER^64^. This approach leverages the CRISPR‐spacer system as a historical record of phage exposure to infer past infection events and calculate the infection rate.

### Growth curve assay

Overnight cultures were diluted to an OD_600_ of 0.1 in fresh LB liquid medium or M9 (2% glycerol) for use as the inoculum. Bacterial suspensions (100 μl per well) were dispensed into 96‐well microtiter plates in triplicate wells and cultured at 37°C. OD_600_ values were recorded per 2 h for enough time to plot growth curves.

### Measurement of pyocyanin production

The mutants of PAO1 strains were grown overnight in LB liquid medium at 37°C with shaking at 220 rpm. Cultures were transformed into fresh PYO medium containing 6 g/l alanine, 0.1 g/l K_2_HPO_4_, 2 g/l MgSO_4_, 1% glycerol, and 0.1 g/l ferric citrate. After 14 h of cultivation, the cells were harvested by centrifugation at 14,000 rpm for 15 min. The supernatants were transferred into a 96‐well plate, on which the OD_695_ of each sample was measured.

### Biofilm production detection

The biofilm production was measured according to a previous study^65^. Briefly, overnight bacterial cultures were transferred to a sterile 10 ml borosilicate tube containing 2 ml of LB medium with the original concentration OD_600_ = 0.1. The cultures grew at 37°C for 14 h without shaking. Adherent biofilms were stained with a 0.1% crystal violet solution for 20 min at room temperature. Excess dye was removed by gently washing with distilled water. After air‐drying, tubes were photographed for visual documentation. Bound crystal violet was subsequently solubilized in 1 ml of 95% ethanol with continuous agitation, and absorbance was measured at 590 nm using a Biotek microplate reader.

### Swarming motility assay

*P. aeruginosa* was grown in LB broth overnight at 37°C. Swarming motility plates contained 8 g/l nutrient broth, 5 g/l glucose, and 0.5% agar. 2.5 μl aliquots were inoculated on swarming plates. Swarming plates were incubated for 16 h at 37°C. Images were captured using a Bio‐Rad imaging system.

### LDH release assay

The LDH release assay was performed using a commercial LDH cytotoxicity detection kit (YEASEN Biotech Co., Ltd.) following the manufacturer's instructions. In brief, *P. aeruginosa* isolates were cultured to a standardized concentration (OD_600_ = 0.4) and used to infect a monolayer of human epithelial cells (A549) at a multiplicity of infection (MOI) of 10:1. The infected cells were incubated for 6 h at 37°C with 5% CO_2_. After incubation, cell culture supernatants were collected. LDH release was measured at OD_490_ (Biotek microplate reader), and the results were calculated as a percentage of the total LDH release from lysed cells. Cytotoxicity levels were expressed as relative LDH release, with higher values indicating stronger cytotoxic effects.

### Plaque assay

The plaque assay was used to evaluate the interactions between different *P. aeruginosa* strains and their prophages. For this, PAO1, PA14, ST463, ST1076, ZR26, and M748 were tested for both their susceptibility to prophages from other strains and their ability to infect other strains. Briefly, bacterial strains were cultured overnight, standardized to an OD_600_ of 2, and mixed with soft agar (0.7%). The mixture was then poured onto LB agar plates with a mixture of prophage and incubated overnight at 37°C. Plaques formed on the bacterial lawns were counted and recorded. An index was calculated based on the ratio of successful infections to resistance, with values closer to 1 indicating stronger infection and resistance capacity, and values closer to –1 indicating higher susceptibility and weaker resistance.

### Mouse model infection assay

The mouse infection model was used to assess the *in vivo* virulence of selected *P. aeruginosa* strains. Pathogen‐free female C57BL/6 mice (6–8 weeks old) were randomly divided into groups (*n* = 5) and inoculated intranasally with 1 × 10^7^ CFU (colony‐forming unit) of each bacterial strain. The mice were monitored for survival per 12 h for up to 60 h post infection.

## AUTHOR CONTRIBUTIONS

**Jiadai Huang**: Formal analysis; investigation; methodology; project administration; validation; visualization; writing—original draft; writing—review and editing. **Kaichao Chen**: Validation. **Miaomiao Xie**: Validation. **Weiyi Shen**: Resources. **Xiaoyang Ju**: Resources. **Yuchen Wu**: Resources. **Jiaping Li**: Resources. **Hongwei Zhou**: Resources. **Yonglu Huang**: Resources. **Yizhou Zhang**: Validation. **Yuanfeng Zhang**: Validation. **Tianmin Li**: Validation. **Letong Xu**: Validation; visualization. **Yue Sun**: Validation. **Fang Chen**: Validation. **Sheng Chen**: Conceptualization. **Xin Deng**: Funding acquisition; investigation; supervision; writing—review and editing. **Rong Zhang**: Conceptualization; funding acquisition; investigation; methodology; resources; writing—review and editing. **Yanyan Hu**: Conceptualization; funding acquisition; investigation; methodology; project administration; resources; supervision; visualization; writing—review and editing.

## ETHICS STATEMENT

Ethical approval was obtained from the Ethics Committee of the Second Affiliated Hospital of Zhejiang University School of Medicine (approval No. 2023‐0611 and 2020‐319). All the specimens were anonymized, and patients were not physically involved in this study. No consent was needed for this study.

## CONFLICT OF INTERESTS

The authors declare no conflict of interests.

## Supporting information


**Figure S1. ST abundance and pan‐genome analysis results of 518 clinically isolated strains from ICU patients and healthy individuals. (A).** ST diversity between healthy and ICU groups. **(B).** The bar chart reveals the ratio comparison of ST abundance between healthy and ICU groups. **(C).** The pie chart shows the distribution of core genes, soft‐core genes, shell genes, and cloud genes. Core genes: present in all genomes; Soft‐core genes: present in at least 95% of the genomes; Shell genes: present in less than 95% but more than 15% of genomes; and Cloud genes: present in less than 15% of the genomes. **(D).** The bubble plot shows the enriched GO terms for core genes. The x‐axis represents the gene ratio, the y‐axis shows the significance, and the bubble size indicates the gene count. **(E‐F).** The bar chart shows GO terms enrichment for cloud genes in the healthy group and the ICU group, respectively.

**Figure S2. Defense and anti‐defense systems' distribution and comparison between ICU and healthy groups. (A).** Diversity of defense systems found in the genomes of *P. aeruginosa* strains from the healthy group, ordered and colored in a gradient of abundance. **(B).** Defense system comparison between ST463, ST1076, and others. **(C‐D).** Diversity of antidefense systems found in the genomes of *P. aeruginosa* strains from the ICU and the healthy group, ordered and colored in a gradient of abundance. **(E).** Fold change of antidefense system abundance in ICU isolates relative to the healthy strains. **(F).** Number of phage donors (other isolates) that successfully lysed each isolate, reflecting host susceptibility. **(G).** Number of recipient isolates that were successfully lysed by phages from each donor, reflecting phage host range.

**Figure S3. AMR gene distribution in the plasmid, chromosome, and prophage. (A‐C).** The heatmap shows the distribution of AMR genes located on plasmid (A), chromosome (B), and prophage (C) across healthy and ICU samples. The rows represent individual samples, while the columns represent different AMR genes.

**Figure S4. Comparison of virulence‐related genes in healthy and ICU isolates. (A).** Distribution of virulence‐related gene counts between ICU and healthy samples. The non‐parametric Kolmogorov–Smirnov test was used for the comparison, showing a non‐significant difference. **(B).** The bar charts visualize the relationships between key virulence genes (*exoS*, *exoU*, *lasR*, *pilA*, *rhsP2*, and *tprA*) and their presence in healthy or ICU samples.

**Figure S5. Quantification of pyocyanin and biofilm production. (A).** Quantification results of PYO production among selected clinical strains. **(B).** Quantification results of PYO production in the *lasR* mutant. **(C).** Quantification results of biofilm production among selected clinical strains. All the error bars used were obtained from at least two biological and technical replicates, and error bars are represented as mean ± standard deviation (SD). The asterisk (*) indicates a statistically significant difference compared to PAO1 (*p* < 0.05, determined by a t‐test).

**Table S1. List of isolated strains**.

**Table S2. List of identified prophages**.

**Table S3. List of AMR genes**.

**Table S4. List of virulence‐related genes**.

## Data Availability

The Illumina sequences generated in this study are deposited and available at NCBI under BioProject number PRJNA1142225, and partially under PRJNA648026, PRJNA767942, and PRJNA716108. Supplementary table [Link], [Link], [Link], [Link] have been put in the Supporting Information section and other materials are available from the corresponding author on request.
