# Supplementary material for: Thirteen‐year genomic analysis (2010–2022) reveals high‐risk ICU Pseudomonas aeruginosa in China
Source: mLife. 2026 Aug 27;5(4):459–72. doi: 10.1002/mlf2.70097 (PMC13519588; doi:10.1002/mlf2.70097)
Supplement: Supplementary file 1 [file MLF2-5-459-s001.pdf]

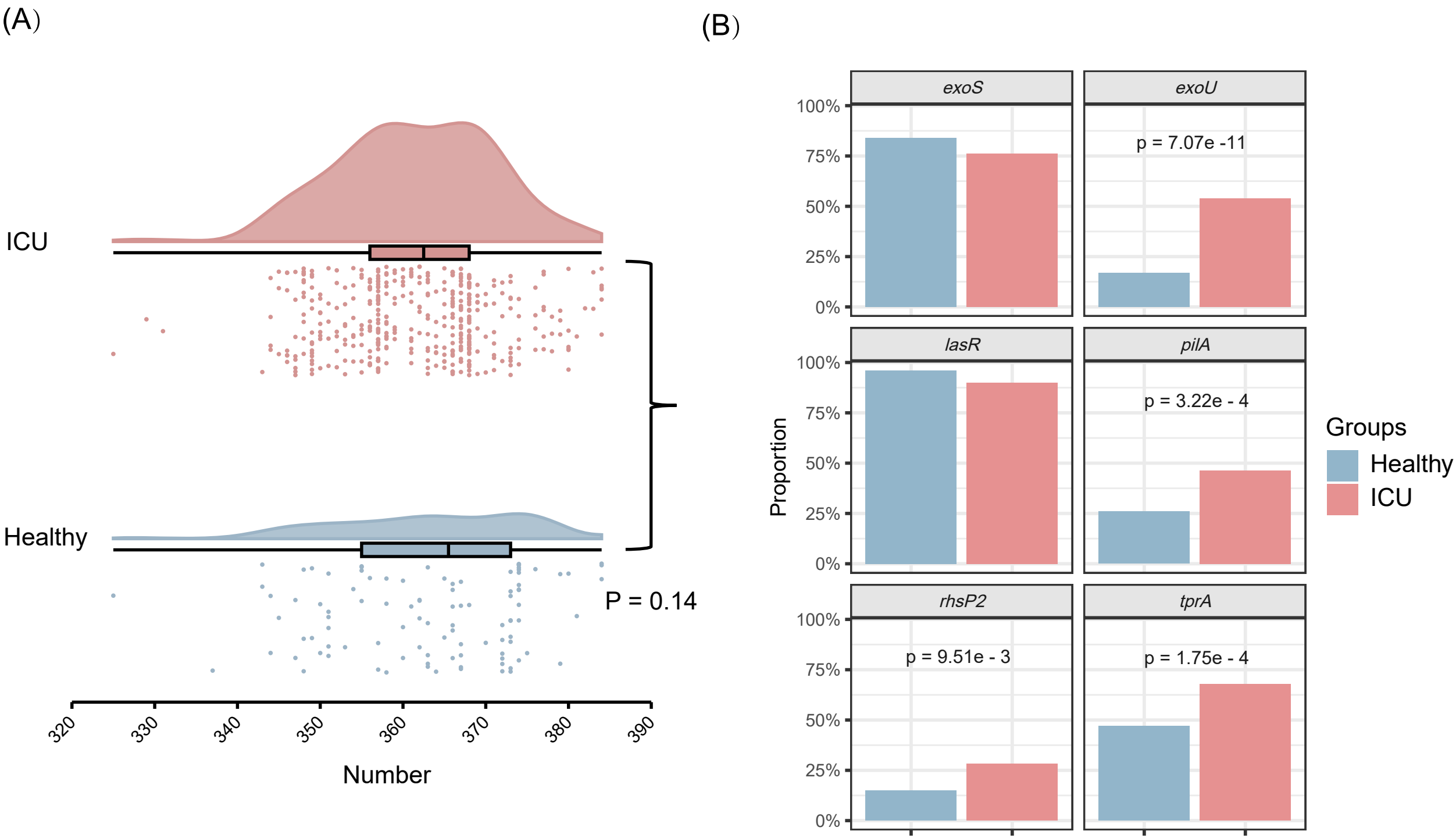

(A)
ICU
Healthy
P = 0.14
320
330
340
350
360
370
380
390
Number
(B)
exoS
exoU
lasR
pilA
rhsP2
tprA
p = 7.07e -11
p = 3.22e - 4
p = 9.51e - 3
p = 1.75e - 4
100%
75%
50%
25%
0%
Proportion
Groups
Healthy
ICU
