# Supplementary material for: Thirteen‐year genomic analysis (2010–2022) reveals high‐risk ICU Pseudomonas aeruginosa in China
Source: mLife. 2026 Aug 27;5(4):459–72. doi: 10.1002/mlf2.70097 (PMC13519588; doi:10.1002/mlf2.70097)
Supplement: Supplementary file 2 [file MLF2-5-459-s003.pdf]

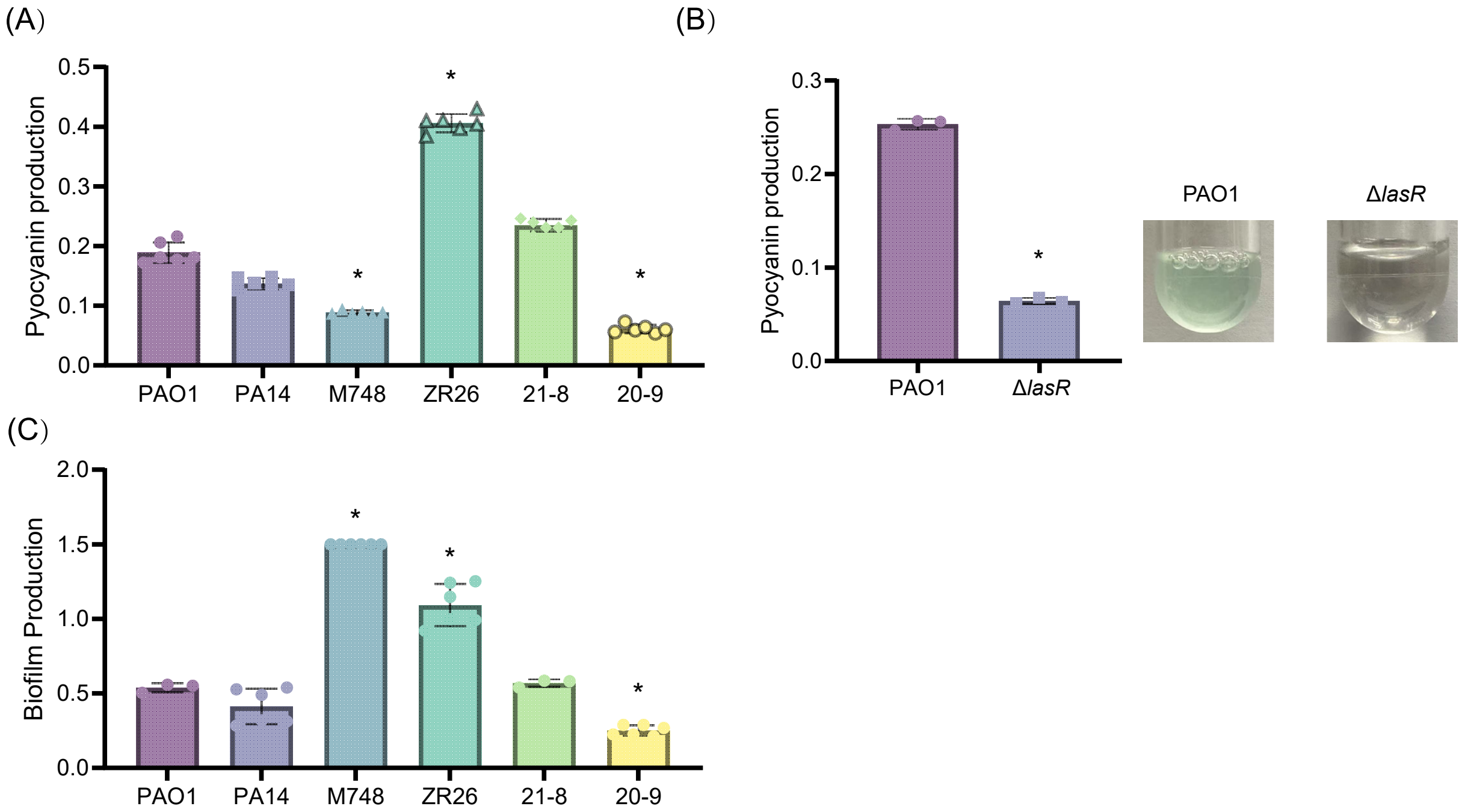
(A)
Pyocyanin production
0.5
0.4
0.3
0.2
0.1
0.0
*
*
*
PAO1
PA14
M748
ZR26
21-8
20-9
(B)
Pyocyanin production
0.3
0.2
0.1
0.0
*
PAO1
ΔlasR
PAO1
ΔlasR
(C)
Biofilm Production
2.0
1.5
1.0
0.5
0.0
*
*
*
PAO1
PA14
M748
ZR26
21-8
20-9
