# Supplementary material for: Thirteen‐year genomic analysis (2010–2022) reveals high‐risk ICU Pseudomonas aeruginosa in China
Source: mLife. 2026 Aug 27;5(4):459–72. doi: 10.1002/mlf2.70097 (PMC13519588; doi:10.1002/mlf2.70097)
Supplement: Supplementary file 3 [file MLF2-5-459-s007.pdf]

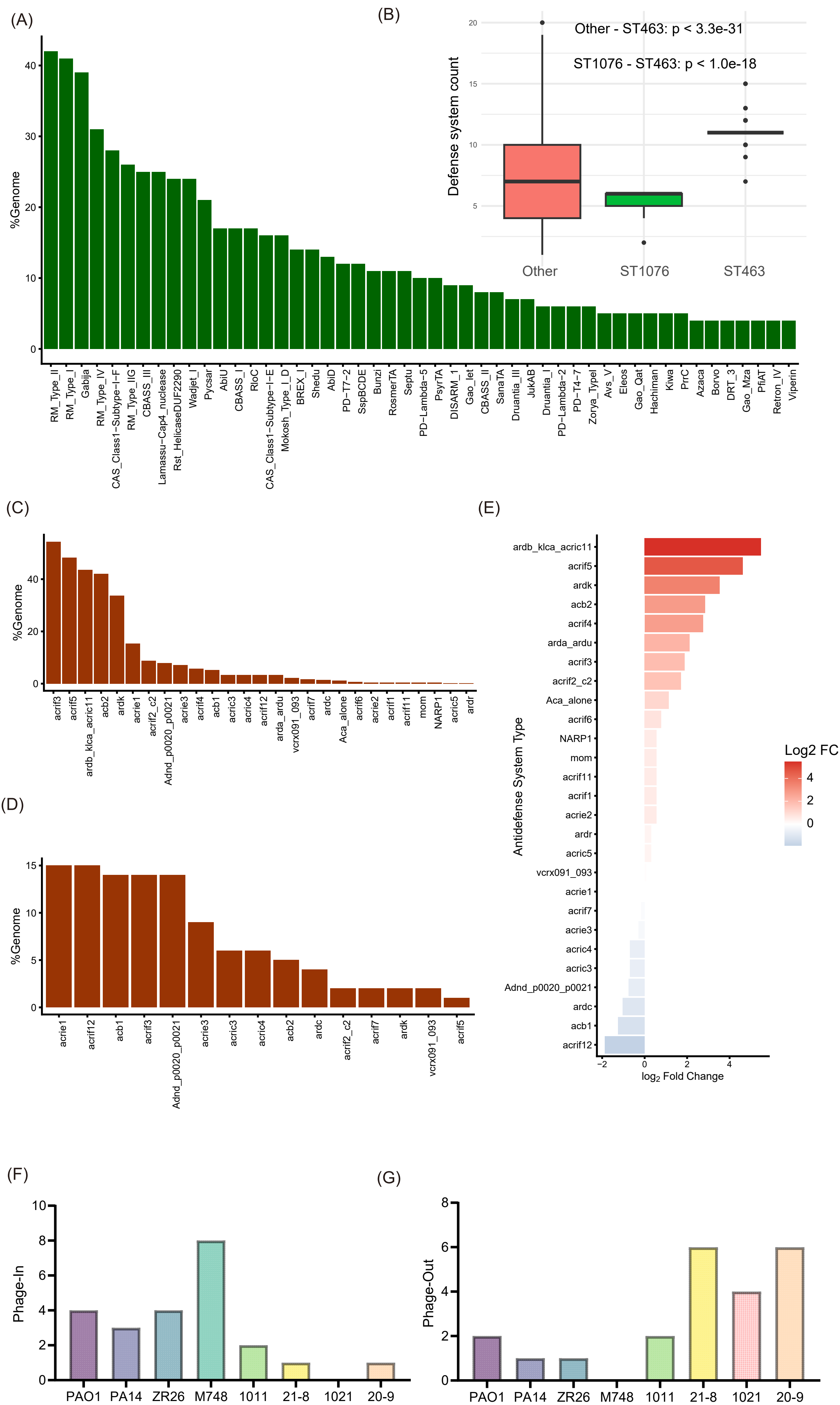
(A)
%Genome
40
30
20
10
0
RM_Type_II
RM_Type_I
Gabija
RM_Type_IV
CAS_Class1-Subtype-I-F
RM_Type_IIG
CBASS_III
Lamassu-Cap4_nuclease
Rst_HelicaseDUF2290
Wadjet_I
Pycsar
AbiU
CBASS_I
RloC
CAS_Class1-Subtype-I-E
Mokosh_Type_I_D
BREX_I
Shedu
AbiD
PD-T7-2
SspBCDE
Bunzi
RosmerTA
Septu
PD-Lambda-5
PsyrTA
DISARM_1
Gao_Iet
CBASS_II
SanaTA
Druantia_III
JukAB
Druantia_I
PD-Lambda-2
PD-T4-7
Zorya_TypeI
Avs_V
Eleos
Gao_Qat
Hachiman
Kiwa
PrrC
Azaca
Borvo
DRT_3
Gao_Mza
PfiAT
Retron_IV
Viperin
(B)
Defense system count
Other - ST463: p < 3.3e-31
ST1076 - ST463: p < 1.0e-18
Other
ST1076
ST463
(C)
%Genome
acrif3
acrif5
ardb_klca_acric11
acb2
ardk
acrie1
acrif2_c2
Adnd_p0020_p0021
acrie3
acrif4
acb1
acric3
acric4
acrif12
arda_ardu
vcrx091_093
acrif7
ardc
Aca_alone
acrif6
acrie2
acrif1
acrif11
mom
NARP1
acric5
ardr
(D)
%Genome
acrie1
acrif12
acb1
acrif3
Adnd_p0020_p0021
acrie3
acric3
acric4
acb2
ardc
acrif2_c2
acrif7
ardk
vcrx091_093
acrif5
(E)
Antidefense System Type
ardb_klca_acric11
acrif5
ardk
acb2
acrif4
arda_ardu
acrif3
acrif2_c2
Aca_alone
acrif6
NARP1
mom
acrif11
acrif1
acrie2
ardr
acric5
vcrx091_093
acrie1
acrif7
acrie3
acric4
acric3
Adnd_p0020_p0021
ardc
acb1
acrif12
log2 Fold Change
Log2 FC
(F)
Phage-In
PAO1
PA14
ZR26
M748
1011
21-8
1021
20-9
(G)
Phage-Out
PAO1
PA14
ZR26
M748
1011
21-8
1021
20-9
