# Supplementary material for: Thirteen‐year genomic analysis (2010–2022) reveals high‐risk ICU Pseudomonas aeruginosa in China
Source: mLife. 2026 Aug 27;5(4):459–72. doi: 10.1002/mlf2.70097 (PMC13519588; doi:10.1002/mlf2.70097)
Supplement: Supplementary file 4 [file MLF2-5-459-s008.pdf]

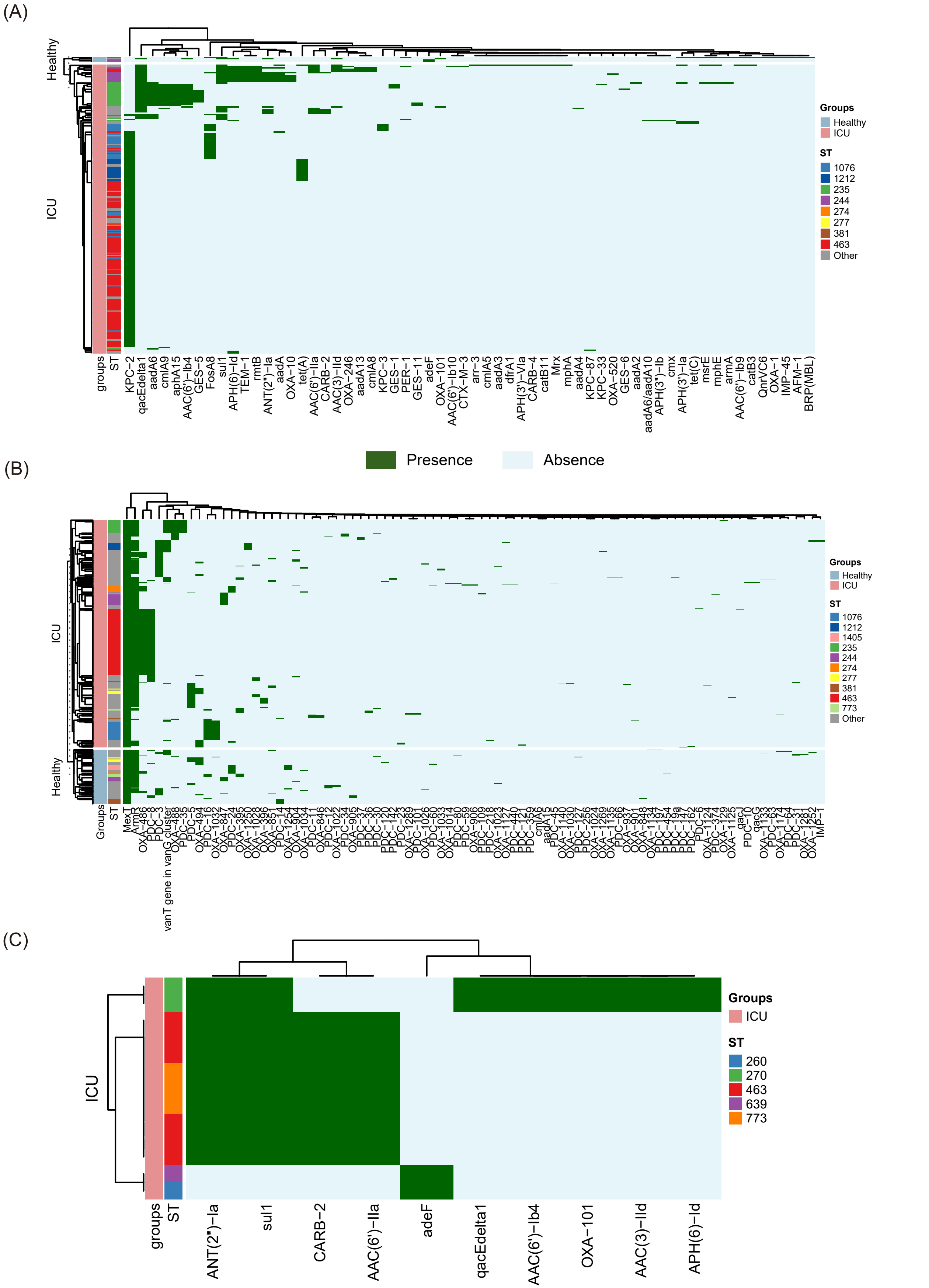
(A)
Healthy
ICU
groups
ST
KPC−2
qacEdelta1
aadA6
cmlA9
aphA15
AAC(6')−Ib4
GES−5
FosA8
sul1
APH(6)−Id
TEM−1
rmtB
ANT(2")−Ia
aadA
OXA−10
tet(A)
AAC(6')−IIa
CARB−2
AAC(3)−IId
OXA−246
aadA13
cmlA8
KPC−3
GES−1
PER−1
GES−11
adeF
OXA−101
AAC(6')−Ib10
CTX−M−3
arr−3
cmlA5
aadA3
dfrA1
APH(3')−VIa
CARB−4
catB11
Mrx
mphA
aadA4
KPC−87
KPC−33
OXA−520
GES−6
aadA2
aadA6/aadA10
APH(3")−Ib
cmx
APH(3')−Ia
tet(C)
msrE
mphE
armA
AAC(6')−Ib9
catB3
QnrVC6
OXA−1
IMP−45
AFM−1
BRP(MBL)
Groups
Healthy
ICU
ST
1076
1212
235
244
274
277
381
463
Other
Presence
Absence
(B)
ICU
Healthy
Groups
ST
vanT gene in vanG cluster
Groups
Healthy
ICU
ST
1076
1212
1405
235
244
274
277
381
463
773
Other
(C)
ICU
groups
ST
ANT(2")−Ia
sul1
CARB−2
AAC(6')−IIa
adeF
qacEdelta1
AAC(6')−Ib4
OXA−101
AAC(3)−IId
APH(6)−Id
Groups
ICU
ST
260
270
463
639
773
