# Supplementary material for: Thirteen‐year genomic analysis (2010–2022) reveals high‐risk ICU Pseudomonas aeruginosa in China
Source: mLife. 2026 Aug 27;5(4):459–72. doi: 10.1002/mlf2.70097 (PMC13519588; doi:10.1002/mlf2.70097)
Supplement: Supplementary file 5 [file MLF2-5-459-s009.pdf]

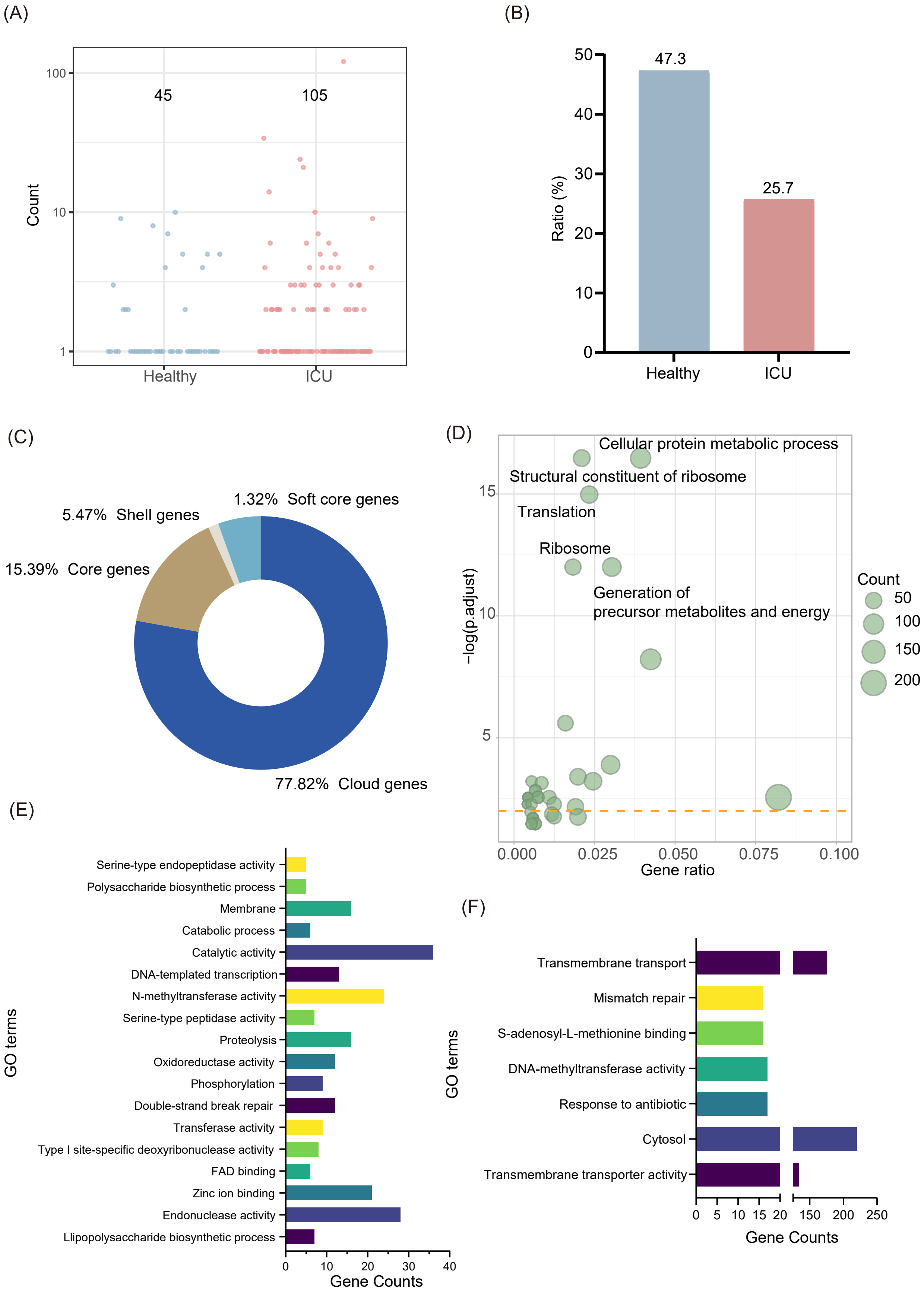
(A)
Count
100
10
1
45
105
Healthy
ICU
(B)
Ratio (%)
50
40
30
20
10
0
47.3
25.7
Healthy
ICU
(C)
1.32% Soft core genes
5.47% Shell genes
15.39% Core genes
77.82% Cloud genes
(D)
Cellular protein metabolic process
Structural constituent of ribosome
Translation
Ribosome
Generation of
precursor metabolites and energy
−log(p.adjust)
15
10
5
Count
50
100
150
200
0.000
0.025
0.050
0.075
0.100
Gene ratio
(E)
GO terms
Serine-type endopeptidase activity
Polysaccharide biosynthetic process
Membrane
Catabolic process
Catalytic activity
DNA-templated transcription
N-methyltransferase activity
Serine-type peptidase activity
Proteolysis
Oxidoreductase activity
Phosphorylation
Double-strand break repair
Transferase activity
Type I site-specific deoxyribonuclease activity
FAD binding
Zinc ion binding
Endonuclease activity
Llipopolysaccharide biosynthetic process
0
10
20
30
40
Gene Counts
(F)
GO terms
Transmembrane transport
Mismatch repair
S-adenosyl-L-methionine binding
DNA-methyltransferase activity
Response to antibiotic
Cytosol
Transmembrane transporter activity
0
5
10
15
20
150
200
250
Gene Counts
